# On-chip constructive cell-Network study (I): Contribution of cardiac fibroblasts to cardiomyocyte beating synchronization and community effect

**DOI:** 10.1186/1477-3155-9-21

**Published:** 2011-05-23

**Authors:** Tomoyuki Kaneko, Fumimasa Nomura, Kenji Yasuda

**Affiliations:** 1 Department of Biomedical Information, Division of Biosystems, Institute of Biomaterials and Bioengineering, Tokyo Medical and Dental University, Tokyo, 2-3-10 Kanda-Surugadai, Chiyoda, Tokyo 101-0062, Japan

## Abstract

**Backgrounds:**

To clarify the role of cardiac fibroblasts in beating synchronization, we have made simple lined-up cardiomyocyte-fibroblast network model in an on-chip single-cell-based cultivation system.

**Results:**

The synchronization phenomenon of two cardiomyocyte networks connected by fibroblasts showed (1) propagation velocity of electrophysiological signals decreased a magnitude depending on the increasing number of fibroblasts, not the lengths of fibroblasts; (2) fluctuation of interbeat intervals of the synchronized two cardiomyocyte network connected by fibroblasts did not always decreased, and was opposite from homogeneous cardiomyocyte networks; and (3) the synchronized cardiomyocytes connected by fibroblasts sometimes loses their synchronized condition and recovered to synchronized condition, in which the length of asynchronized period was shorter less than 30 beats and was independent to their cultivation time, whereas the length of synchronized period increased according to cultivation time.

**Conclusions:**

The results indicated that fibroblasts can connect cardiomyocytes electrically but do not significantly enhance and contribute to beating interval stability and synchronization. This might also mean that an increase in the number of fibroblasts in heart tissue reduces the cardiomyocyte 'community effect', which enhances synchronization and stability of their beating rhythms.

## Background

Cardiomyocytes make up more than half the volume of normal heart tissue and play a role in the pumping of blood. Most of the other, non-beating, cells in the heart is the fibroblasts forming the cardiac skeleton and providing the mechanical scaffold for cardiomyocytes. Fibroblasts are also more plentiful in diseased hearts than healthy hearts, so one must consider the possibility that electrical coupling between fibroblasts and cardiomyocytes plays a role in arrhythmogenesis [1-3]. It has, in fact, been shown in cell culture that the electrical coupling of fibroblasts can propagate the contraction among cardiomyocytes [4-7]. However, the conventional *in vitro *experiments of cardiomyocyte-fibroblast networks were examined by the randomly connected cells in the cultivation dishes [8-10]. Hence it is difficult to measure the time course change of particular cells before/after connection formation. To overcome this problem, one of the ways is to use microstructures to fix their positions, distances and interactions.

The principles of patterned growth of cultured cardiomyocytes were pioneered in the early 70s, and in the early 90s the introduction of photolithographic techniques resulted in a method that could be used to define the patterns of cardiomyocytes grown in primary culture [11]. That method did not work well with fibroblasts, however, because they tended to adhere and extend to the photoresist, and hence the patterned structure could not control the single-cell level control of their positions. Moreover, although the interaction of the heterogeneous cell types was studied using this method, those studies were done with clusters rather than isolated single cells [5,12]. Hence, the measurement of electrical coupling between fibroblasts and cardiomyocytes was not considered as the single fibroblast's electrical coupling function. To overcome these problems, we developed an agarose microchamber system by using the photothermal etching method [13,14]. This system has been used to control the network patterning of neurons [15-17] and to control the connections of cardiomyocytes [18,19]. Using that system to examine the contribution of the 'community effect' to the stability of the beating in the homogeneous cardiomyocyte networks [20,21], we found that the beating of an *in vitro *community (network) comprising nine cells is as stable as the beating of the heart, that the rhythms of two isolated cells became synchronized after the cells made physical contact with each other, and that the synchronized rhythm of those two cells was the more stable one rather than the faster one [22]. We did not, however, examine the role of the community effect in heterogeneous cell networks, especially in cardiomyocytes.

In this study, we have examined the single-cell-based minimum heterogeneous network of cardiomyocytes and fibroblasts on a chip, measured the time course of changes in the stability of the synchronization of two cardiomyocytes connected by a fibroblast, analyzed the contributions of cardiac fibroblasts to the synchronization of cardiomyocyte beating, and discussed the effect of the fibroblast population in heart tissue on the 'community effect' of cardiomyocyte network synchronization.

## Methods

### Cardiomyocyte and cardiac fibroblast isolation and culture

Embryonic mouse cardiomyocytes were isolated and purified using a modified version of a method described in Ref. [22]. All animal protocols and experiments were approved by the Institutional Animal Care and Use Committee of Tokyo Medical and Dental University (Ethical Approval Number: 0110091A). In brief, the cardiomyocytes were isolated from 13-to-14-day-old ICR mouse embryos (Saitama Experimental Animals Supply, Japan). After the embryos were rapidly removed from a mouse anesthetized with diethyl ether, the hearts of the embryos were removed and washed with phosphate-buffered saline (PBS: 137 mM NaCl, 2.7 mM KCl, 8 mM Na_2_HPO_4_, 1.5 mM KH_2_PO_4_, pH 7.4) containing 0.9 mM CaCl_2 _and 0.5 mM MgCl_2 _to induce heart contraction and remove corpuscles. The hearts were then transferred to PBS without CaCl_2 _and MgCl_2 _and the ventricles were separated from the atria, minced into 1-mm^3 ^pieces with fine scissors, and incubated at 37°C for 30 minutes in PBS containing 0.25% collagenase (Wako, Osaka, Japan) to digest the ventricular tissue. After this procedure was repeated twice, the cell suspension was transferred to Dulbecco's modified Eagle's medium (DMEM: Invitrogen, Carlsbad, CA, USA) supplemented with 10% fetal bovine serum, 100 U/ml penicillin, and 100 μg/ml streptomycin at 4°C. The cells were filtered through a 40-μm-nylon mesh and then centrifuged at 180 g for 5 minutes at room temperature. After the cell pellet was resuspended in a supplemented DMEM, 100 μl of the suspension (diluted to a final concentration of 1.0 × 10^5 ^cells/ml) was plated onto a 35-mm dish and the individual cardiomyocytes were picked up one by one using a micropipette (Tip diameter: 20 μm) with micromanipulation system (CellTramAir and Micromanipulator 5171 [Eppendorf, Hamburg, Germany]) and put into the microchambers in the cultivation dish. Cell-handling pipettes (inner diameter: 0.03 mm) were fabricated by pulling glass capillaries (outer diameter: 1 mm; GD-1, Narishige, Japan) with a puller (PC-10, Narishige Japan), and cutting, and fire polishing the cut end of the tubes with a microforge (MF-900, Narishige, Japan). The inner and outer surfaces of cell-handling pipettes were coated with sigmacote (SL-2; Sigma-Aldrich, MO, USA) by evaporation at room temperature in order to prevent cell adhesion onto the pipettes. For distinguishing target cardiomyocytes, we have checked their smooth surfaces and their sizes as indexes. Then, we cultivated the cells in the microchambers and we chose the microchambers in which two cardiomyocytes were successfully beating in both of chambers for the further experiments.

The fibroblasts were identified by their fast cell division and extension speed just after cultivation. Cardiac fibroblasts were obtained from the remaining cells after the cardiomyocytes isolation procedure. The obtained cells were cultured on a tissue-cultured dish more than 5 passages in supplemented DMEM. As the fibroblasts increased and formed a monolayer on the dish, the number of cardiomyocytes in cultivated cells was substantially decreased. Cardiac fibroblasts were harvested with 0.25% trypsin/ethylenediaminetetraacetic acid (EDTA: Invitrogen, Carlsbad, CA, USA) and selected by their rough shape and size after 20 min of suspension cultivation. Using a micropipette, cardiac fibroblasts were picked up and put into the chosen microchambers where both of two cardiomyocytes was beating successfully.

### Image analysis

The spontaneous contraction rhythm of cultured cardiomyocytes was evaluated by a video-image recording method as described previously [20-22]. Briefly, images of beating cardiomyocytes were acquired with a charge-coupled device (CCD) camera attached to a phase contrast microscope, recorded by a video cassette recorder (VCR), and analyzed using a video capture system on a personal computer. From each image a small region where intensity changed considerably with contraction was selected and the average signal intensity of the selected area was digitized by a personal computer. Temporal variations of average signal intensity in the selected area correspond to the contraction rhythm of the cardiomyocytes.

### Patch-clamp measurement

Double whole-cell patch-clamp recordings were achieved with multiclamp 700B (Axon Instruments) patch-clamp amplifier. The transmembrane potential was recorded using the whole cell recording mode of the patch-clamp technique. Patch pipettes (6-7MΩ resistance) were pulled from glass capillary tubes and filled with pipette solution (in mM: 100 K-gluconate, 40 KCl, 4 Na-ATP, 1 MgCl2, 0.5 EDTA, and 5 HEPES, with pH adjusted to 7.4 with KOH). The bath solution contained (in mM) 145 NaCl, 4 KCl, 1 CaCl2, 1 MgCl2, 1 glucose, and 10 HEPES, with pH adjusted to 7.4 with NaOH. For data acquisition and analysis Clampex9.2 software (Axon Instruments) was used. We measured the time lag between two action potentials at 0 mV.

### Statistics

Data are given as mean ± SD. Data sets were compared using the Student t test (2-tailed), and differences were considered significant at P < 0.001.

### Immunofluorescence staining

After the measurements, the preparations were washed with PBS, fixed with 4% paraformaldehyde for 15 minutes at room temperature, and permeabilized in 0.1% Triton X-100 for 15 min. Thereafter, they were incubated at room temperature for 1 hour with blocking buffer (PBS containing 1% BSA) before being exposed for 2 hours to the primary antibodies (mouse monoclonal antibody to heavy chain cardiac myosin, abcam, Tokyo, Japan, and rabbit polyclonal antibody to connexin-43, Sigma-aldrich, St. Louis, MO, USA) dissolved in blocking buffer. Finally, the preparations were washed and incubated for 1 hour at room temperature with secondary antibodies (Alexa Fluor 488, goat anti-mouse IgG, and Alexa Fluor 546, goat anti-rabbit IgG, Molecular probes, Eugene, OR, USA). To visualize the nuclei, cells were counterstained with Hoechst 33342 for 30 min at room temperature. The preparations were imaged on an inverted microscope equipped for epifluorescence (IX-70, Olympus, Tokyo, Japan) using cooled CCD camera (ORCA-ER, Hamamatsu photonics, Shizuoka, Japan).

## Results and discussion

### On-chip single-cell-based cell observation system using an agarose microchamber

**Figure 1:**
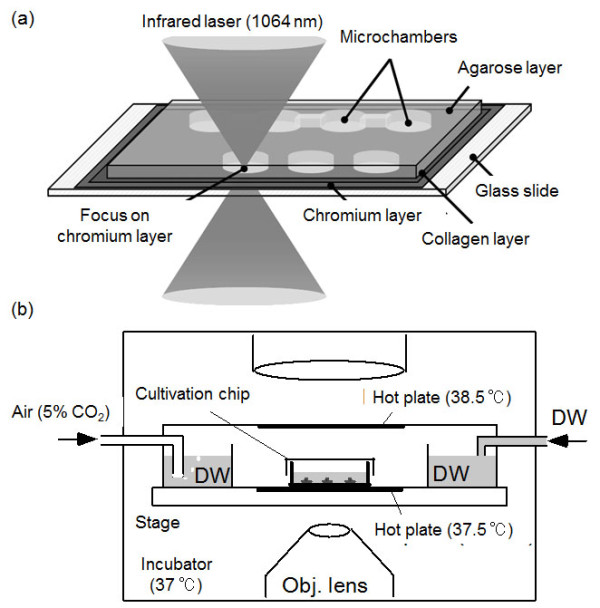
**On-chip single-cell-based cell culture system using agarose microchambers**. (a) Making of microchambers. Collagen was applied to the chromium-coated glass slides in order to improve the attachment of the cells. After the slides were spin-coated with agarose, microchambers and channels connecting them were formed using a 1064-nm infrared laser beam. (b) On-chip single-cell-based cultivation and observation system.

Agarose microchambers were made using a modified version of a method described previously [13-22]. In brief, the attachment of cardiomyocytes to the bottom of the microchambers was improved by coating the 5-nm chromium layer on a glass slide with type І collagen (Nitta gelatin, Osaka, Japan) before depositing 50 μm of a 2% (w/v) agarose solution (ISC BioExpress, GenePure LowMelt: melting temperature 65°C) on it by spin coating at 4,000 rpm for 30 sec (Spincoater 1H-D7, Mikasa, Tokyo, Japan). After the agarose was hardened into a gel by keeping the slide in a refrigerator at 4 °C, a 1064-nm infrared laser beam (Nd: YAG laser; PYL-1-1064-M, IPG Laser GmBH, Germany) focused on the chromium layer was used to melt three-microchamber linear arrays in the agarose layer. Because the 1064-nm infrared laser beam is permeable to water, thin stable chromium bottom layer was used for absorption of the 1064-nm laser for further μm-order spot heating of a portion of agarose layer to form microstructures. A microscope observation was used to confirm that the melting had occurred, and then either the heating was continued until the microchamber reached the desired size or the heating position was shifted to create a channel connecting that microchamber with an adjacent one (Figure 1(a)). As the focused beam was moved, parallel to the chip surface, from one microchamber to another the agarose adjacent to the heated chromium melted and diffused into water, forming a channel. Individual cardiomyocytes were micropipetted into the end microchambers and cultured there at 37°C in a humidified atmosphere (95% air and 5% CO_2_) in a cell culture container (INU-ONIG; Tokai Hit, Shizuoka, Japan) mounted on a phase contrast microscope (Figure 1(b)).

### Formation of single-cell-based cardiomyocytes and fibroblast network model

**Figure 2:**
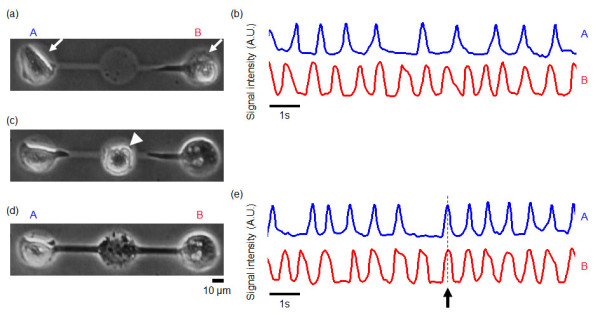
**Interaction through a cardiac fibroblast of two cardiomyocytes with different rhythms**. (a) A phase-contrast image of two cardiomyocytes (white arrows) with different beating rhythms cultured in microchambers A and B (48 hours after cultivation started). (b) Time course of two cardiomyocytes' beating rhythm before synchronization. (c) Using glass micropipette, single cardiac fibroblast (white arrowhead) was set at the center of three lined-up microchambers (72 hours after cultivation started). (d) The two cardiomyocytes were connected through single cardiac fibroblast (6 hours after re-cultivation started, i.e., 76 hours after cultivation started). (e) Time course of beating rhythms of cardiomyocytes cultured in microchambers A and B after synchronization. Dashed line shows time that synchronization occurred.

For the precise evaluation of cell-to-cell connection of cardiomyocytes and fibroblasts quantitatively, especially to compare the characteristics before and after their connection to be formed and to control their spatial arrangements and their distances, on-chip single-cell-based microfabrication and cultivation technology was useful. We cultivated single fibroblasts to connect isolated two cardiomyocytes cultivated in both sides of three lined-up microchambers so that we could see how two cardiomyocytes with different beating rhythms synchronized their rhythms through fibroblasts. First, the two single cardiomyocytes were cultured in the two microchambers at the ends of a three-microchamber array, and Figure 2(a) shows the cell growth 48 hours after cultivation started. At this time the two cardiomyocytes did not contact each other and their beating rhythms were independent and uncorrelated even the two cells were obtained from same tissue sample (Figure 2(b)). Then, to connect the two cardiomyocytes through a fibroblast, 72 hours after starting the cultivation we put a single fibroblast into the center microchamber (Figure 2(c)) and continued the cultivation. Finally, as shown in Figure 2(d), 6 hours later, a cardiomyocyte-fibroblast-cardiomyocyte network had formed as a result of fibroblast elongation and attachment to the two cardiomyocytes. The cardiomyocytes connected by the fibroblast then synchronized their beating rhythm (an arrow in Figure 2(e)). It should be noted that, as in the synchronization of homogeneous cardiomyocyte networks [22], the synchronized rhythm was not intermediate between the individual rhythms but was one of them. As shown in Figure 2(e), for example, during the synchronization of the independent rhythms of cells A and B, the beating of cell A stopped and then restarted in synchrony with the beating of cell B.

These results show that cardiomyocyte-fibroblast connections can couple a fibroblast and two asynchronously beating cardiomyocytes into a three-cell network in which the rhythms of the cardiomyocytes are synchronized and that the process of establishing a synchronous state can be observed continuously at the single-cell level.

**Figure 3:**
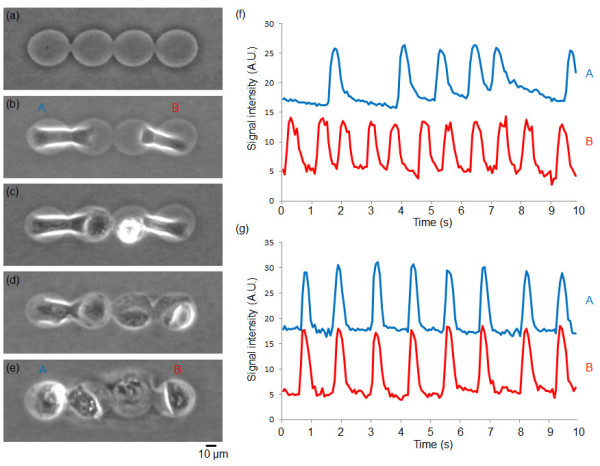
**Interaction of two cardiomyocytes through two cardiac fibroblasts**. (a) Four agarose microchamber array fabricated on the cultivation chip. (b) Two cardiomyocytes cultivated in both sides of four microchambers (A, B) (micrograph image acquired 24 hours after cultivation started). (c) After the confirmation of two cardiomyocytes' beating, two fibroblasts were put into the two center microchambers (micrograph, 1 h after fibroblast cultivation started). (d) Confirmation of fibroblasts because of their fast elongation ability (2 h after (c)). (e) Synchronization of two cardiomyocytes through two fibroblasts (1 day after fibroblasts' addition). (f) Time course of beating rhythms of cardiomyocytes cultured in microchambers A and B before synchronization at (b), and (g) after synchronization at (e).

Figure 3 showed another example of four cell network formation on a chip. Just same as three cell network model, first, two cardiomyocytes were settled both ends of four lined-up microchambers (A and B in Figure 3(b)). After the confirmation of their beating, two cardiac fibroblasts were settled in the remaining two center microchambers (Figure 3(c)), and finally these four cells were connected, and synchronized (Figures 3(e) and 3(g)).

### Synchronization of two cardiomyocyte beating through a fibroblast

**Figure 4:**
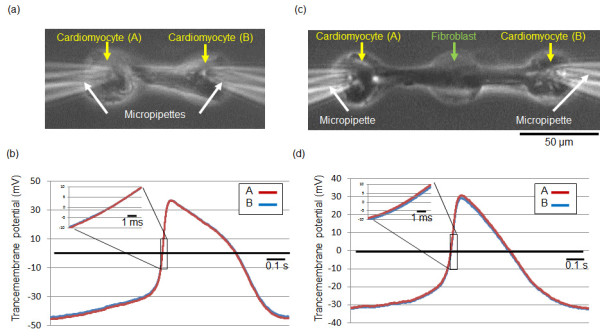
**Electrophysiological measurement of synchronization of two cardiomyocytes with/without a fibroblast between them**. (a) A phase-contrast image of two cardiomyocytes (A, B; yellow arrows) and two micropipettes (white arrows) for electrophysiological recording. Two cardiomyocytes were connected through the channel fabricated in the agarose layer on a chip. (b) Time course of two cardiomyocytes' beating action potentials. (c) A phase-contrast image of a lined-up two cardiomyocytes (C, D; yellow arrows) and single fibroblast (green arrow) network (72 hours after cultivation started). Two micropipettes (white arrows) were put on two cardiomyocytes for electrophysiological recording. Two cardiomyocytes were connected through the channel fabricated in the agarose layer on a chip. (d) Time course of two cardiomyocytes' beating action potentials connected by a fibroblast. In detail, see Table 1.

In Figures 2 and 3, the synchronization of two cardiomyocytes was observed by optical measurement of those cells' displacements. Then we have evaluated the electrical connection of two cardiomyocytes with/without fibroblast between them using double whole-cell patch-clamp recordings for studying the characteristics of connections quantitatively. Figures 4(a) and 4(b) showed an example of two cardiomyocyte network measurement and the results of electrical connections of two cardiomyocytes. Figures 4(c) and 4(d) also showed an example of two cardiomyocyte network connected through a cardiac fibroblast. As shown in the graph, slight delay of electrical potential change was observed when the fibroblast was added between two cardiomyocytes.

Table 1 is a summary of a series of two cardiomyocytes' delay times. In this experiment, applying the advantage of our agarose microchamber cultivation method, we control the distances of cells strictly. First, the direct connections of two cardiomyocytes (CM-CM) with 60 μm distance showed less than 0.1 ms delay of propagation (average: 0.055 ms) and average conduction velocity of 1.3 m/s. In contrast, the delay time of propagation in two cardiomyocytes connected by a fibroblast (CM-F-CM) increased to 0.7 - 6.0 ms (average: 3 ms), and was a magnitude slower than the direct connection of two cardiomyocytes both in the 120 μm and 180 μm distance models, i.e., 60 μ m and 90 μ m distances between cardiomyocyte and fibroblast respectively. Average conduction velocity with 120 μm distance (CM-F-CM) was 0.08 m/s. There are significantly difference (P < 0.001) between conduction velocity of CM-CM and one of CM-F-CM. Moreover, when we arranged two fibroblasts between two cardiomyocytes (CM-F-F-CM) with 60 μm distances between neighboring cells, the propagation delay increased to 11 ms, and was obviously slower than that of single fibroblast connection model.

**Table 1:** Electrical connection of two cardiomyocytes

Connection type*^1^	Distance (μm)*^2^	Delay time (ms)*^3^	Velocity (m/s)*^4^	N*^5^
CM-CM	60	0.031 ± 0.03	1.8 ± 0.8	12
CM-CM	60	0.059 ± 0.02	1.3 ± 0.7	24
CM-CM	60	0.063 ± 0.03	1.3 ± 0.7	20
CM-CM	60	0.068 ± 0.008	0.90 ± 0.1	60

CM-F-CM	120	0.67 ± 0.03	0.18 ± 0.008	93
CM-F-CM	120	1.5 ± 0.2	0.080 ± 0.01	39
CM-F-CM	120	3.8 ± 0.3	0.032 ± 0.003	95
CM-F-CM	120	6.0 ± 0.6	0.020 ± 0.002	48

CM-F-CM	180	0.91 ± 0.4	0.23 ± 0.08	29
CM-F-CM	180	2.2 ± 0.3	0.085 ± 0.01	50

CM-F-F-CM	180	11 ± 0.4	0.016 ± 0.0006	6

^*1^Connection type: CM-CM means two cardiomyocytes directly connection. CM-F-CM means two cardiomyocytes connected by one fibroblast. CM-F-F-CM means two cardiomyocytes connected by two fibroblasts.

^*2^Distance: interval of the tips of micropipettes.

^*3^Delay time: interval between the action potentials of two cardiomyocytes at 0 mV (mean ± SD).

^*4^Velocity: conduction speed (distance/delay time).

^*5^N: sampling number of spikes.

The above results showed that the fluctuations in CM-F-CM samples having same 60 μm distances were larger than the difference of fluctuations between CM-F-CM samples having 60 μm distances and 90 μm distances, and also showed the addition of fibroblast significantly contributed to delay the propagation. These results indicated that the delay of propagation was mainly occurred by the increase of number of fibroblasts, not by the extension of fibroblasts.

### Community effect in cardiomyocyte networks coupled through fibroblasts

Then, we used this heterogeneous cardiomyocyte-fibroblast coupling system to examine the tendency of the stability of interbeat intervals and beating rhythm fluctuation of two cardiomyocytes before and after their synchronization through a fibroblast. In our previous study of using homogeneous (i.e., direct) coupling of two cardiomyocytes [22], the tendency of the synchronization was simply explained by saying that the synchronization of two cardiomyocytes was caused by the more unstable cell (the one with the more variable beating intervals) following the more stable cell. Such fluctuation reduction tendency was more obvious when the number of cardiomyocytes in the network increased, and we call this phenomenon as "community effect" of synchronization [21-23].

Evaluating the mechanism of community effect, we also should compare the heterogeneous cell networks against the homogeneous cell networks. Hence we have examined the synchronization of the two-cardiomyocyte network having a fibroblast connection, and found two types of tendencies of the fluctuation of beating intervals before and after synchronization.

**Figure 5:**
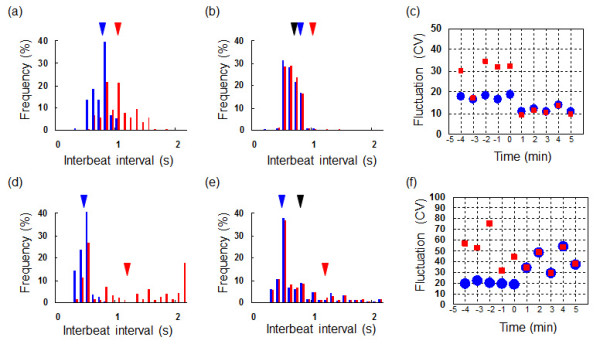
**Distribution of interbeat intervals of two cardiomyocytes coupled through a cardiac fibroblast, and changes in the mean beating rhythm fluctuation before and after synchronization**. (a)(d) Distribution of interbeat intervals before synchronization. Blue and red bars show the frequency (%) of each interbeat interval for two cardiomyocytes, and blue and red arrowheads indicate the mean values for each. (b)(e) Distribution of interbeat intervals after synchronization. Blue and red arrowheads indicate the before-synchronization mean values for the same two cardiomyocytes whose data are shown in (a) and (d) respectively, and the black arrowheads show the mean value for the synchronized cardiomyocytes. (c)(f) Beating rhythm fluctuation (coefficient of variation, CV) in 1-min intervals before and after synchronization. Blue filled circles and red filled squares show mean values for the same two cardiomyocytes whose data are shown in (a) and (d) respectively. Results for two kind pairs are shown in (a)-(c) for fluctuation CV decrease and (d)-(f) for those increase. In detail, see Table 2.

The first type was the tendency of fluctuation reduction caused by synchronization, which is same tendency seen in a network formed by the direct connection of two cardiomyocytes. As shown in Figure 5, in this case, the two cells having interbeat intervals of 0.78 s and 1.1 s before synchronization (Figure 5(a)) had made a synchronized interbeat interval of only 0.65 s after synchronization (Figure 5(b)). The fluctuation of synchronized network became smaller than either of the two initial fluctuations (Figure 5(c)).

In contrast, the second type was the tendency of fluctuation increase caused by synchronization, which was not occurred in the cardiomyocyte network. In this case, the two cells having two interbeat intervals of 0.48 s and 1.2 s before synchronization (Figure 5(d)) had a mean interbeat interval of 0.79 s after synchronization (Figure 5(e)), and the fluctuation of the synchronized network was greater than that of the cell that had the lower fluctuation before the synchronization (Figure 5(f)).

Tables 2 and 3 showed the results of synchronization of two cardiomyocytes connected through a fibroblast. All the fluctuation CV decrease samples (Table 2) showed reduction of fluctuation from both of CV's before synchronization regardless of the tendencies of synchronized interbeat intervals (IBIs) formation. However, the fluctuation CV increase samples (Table 3) showed the CVs of synchronized cardiomyocytes were larger than one of the smaller CV cardiomyocytes. That is, the improvement of fluctuation by network formation was not observed.

**Table 2:** CV down group of interbeat interval (IBI) and fluctuation (CV) of two cardiomyocytes networks connected by a fibroblast

CV down	Before		After	Before		After
**Sample No**.	IBI (Left)	IBI (Right)	IBI	vCV (Left)	CV (Right)	CV
1	0.78 ± 0.13	1.1 ± 0.32	0.65 ± 0.08	17	29	12
2	2.4 ± 3.1	1.2 ± 0.54	1.0 ± 0.34	130	45	34
3	0.63 ± 0.25	2.3 ± 1.1	1.0 ± 0.34	39	49	34
4	5.0 ± 6.9	0.43 ± 0.12	0.59 ± 0.10	140	28	17
5	2.3 ± 1.9	0.57 ± 0.09	0.55 ± 0.08	84	16	15

**Table 3:** CV up group of interbeat interval (IBI) and fluctuation (CV) of two cardiomyocytes networks connected by a fibroblast

CV up	Before		After	Before		After
**Sample No**.	IBI (Left)	IBI (Right)	IBI	CV (Left)	CV (Right)	CV
1	0.48 ± 0.10	1.2 ± 0.62	0.79 ± 0.32	20	52	40
2	1.2 ± 0.26	0.62 ± 0.10	0.73 ± 0.14	22	16	19
3	0.52 ± 0.10	6.1 ± 11	0.78 ± 0.32	19	180	41
4	1.9 ± 1.6	0.86 ± 0.15	1.6 ± 0.59	82	18	37
5	2.6 ± 1.4	0.57 ± 0.13	3.8 ± 1.8	53	22	47
6	22 ± 17	1.7 ± 0.80	6.0 ± 4.4	78	47	74

We also have checked the phenomenon of three cardiomyocyte networks (CM-CM-CM) for the confirmation (Table 4), and found all of three samples were categorized into the fluctuation CV decrease samples same as we have reported previously [23]. Hence, the fluctuation CV increase samples should be caused by the fibroblast, which is connecting two cardiomyocytes.

**Table 4:** Interbeat interval (IBI) and fluctuation (CV) of three cardiomyocytes networks.

	Before		After	Before		After
**Sample No**.	IBI (Left)	IBI (Right)	IBI	CV (Left)	CV (Right)	CV
1	0.36 ± 0.08	0.48 ± 0.12	0.42 ± 0.06	22	25	14
2	0.83 ± 0.20	0.78 ± 0.17	0.76 ± 0.15	24	21	20
3	0.46 ± 0.15	1.3 ± 0.53	1.3 ± 0.19	33	42	15

These results indicate that the interbeat interval after the synchronization of two cardiomyocytes connected by a fibroblast is not same as that after the synchronization of two cardiomyocytes directly connected to each other [22], and the tendency of community effect seems to be suppressed when the cardiomyocytes are heterogeneously coupled through a fibroblast. Since the gap junctions between fibroblasts and cardiomyocytes are smaller than those between pairs of cardiomyocytes [4,5], the suppression of this tendency might be due to the lower electrical conductivity. This suggests that the community effect in the synchronization of cultured cardiomyocytes--that is, the enhanced synchronization seen with larger communities--will be most evident in homogeneous cardiomyocyte clusters.

### Time course of stability of cardiomyocyte networks coupled through fibroblasts

**Figure 6:**
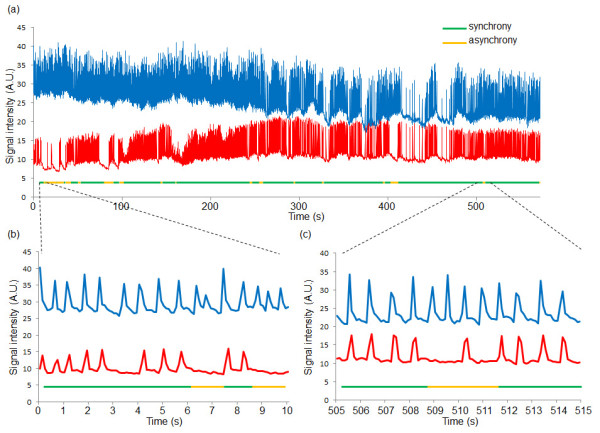
**Time-course of beating synchronization of two cardiomyocytes connected through a cardiac fibroblast**. (a) 10 min of beating rhythms of two cardiomyocytes (blue line and red line). The line under the beating rhythms indicate the condition of their synchrony, i.e., green line indicates synchronized condition, whereas yellow line indicates asynchronized condition. (b) Magnified graph of Figure (a) from 0 to 10 s, and (c) from 505 to 515 s.

Investigating the time course of the stability of synchronization, we also checked the possibility of occurrence of asynchronization after their synchronization accomplished. Two cardiomyocytes for long-term observation were cultured in the chambers at the ends of a three-microchamber array in which a fibroblast was cultured in the center chamber. The fibroblast grew and extended through the narrow channels connecting adjacent chambers until it was attached to the two cardiomyocytes (Figure 6(a)), which then started to synchronize. After the synchronization accomplished, however, the beating of the two cardiomyocytes later became asynchronous (Figures 6(b) and 6(c)).

This after-synchronization asynchronization was not seen in our earlier study using directly connected cardiomyocytes [22]. It also should be noted that this asynchronization phenomenon was observed in all the above two types of fibroblast-cardiomyocyte synchronization.

For the confirmation of long term cultivation, we also have observed three of the CM-F-CM samples continuously for 6 h, and found that their fluctuation (CV) decreased gradually during cultivation, and no asynchronization occurrence was observed when we observed them 6 h after the synchronization accomplished (Table 5). The result indicates that the asynchronization was temporal phenomenon and finally they synchronized completely within 6 h during long term cultivation.

**Table 5:** Long term observation of interbeat intervals and fluctuation of two cardiomyocytes connected by a fibroblast

Before					After 0h		After 0h		After 6h	
**Sample No**.	IBI (Left)	IBI (Right)	CV (Left)	CV (Right)	IBI	CV	IBI	CV	IBI	CV
1	0.77 ± 0.23	0.67 ± 0.11	30	16	0.56 ± 0.08	15	0.30 ± 0.06	20	0.27 ± 0.04	14
2	0.55 ± 0.23	0.81 ± 0.14	42	17	0.74 ± 0.10	14	0.58 ± 0.08	14	0.59 ± 0.07	12
3	0.48 ± 0.18	0.49 ± 0.12	37	25	0.47 ± 0.11	27	0.40 ± 0.05	12	0.37 ± 0.04	11

**Figure 7:**
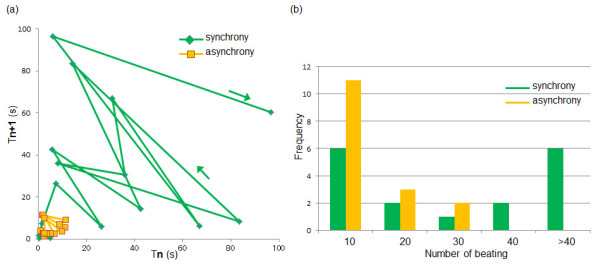
**Tendency of synchronization and asynchronization of cardiomyocyte network connected through a fibroblast**. (a) Logistic map of synchronized intervals and asynchronized intervals, (b) frequency of the synchronized condition length or asynchronized condition length of sample shown in Figure 6.

Figure 7 showed the tendency of synchronization and asynchronization of cardiomyocyte network connected through a fibroblast. Figure 7(a) is the logistic map of neighboring synchronized periods and the asynchronized periods replotted from the data shown in Figure 6. If the neighboring periods have any kind of correlations, the results (plotted dots) should show some pattern on the map. The results indicated that 1) the tendency of the length of the synchronized period increased gradually depending on the cultivation time, whereas the length of asynchronized periods did not changed, 2) both of the length of the synchronized period and the length of asynchronized periods showed no obvious correlation between neighboring periods (i.e., no hysteresis).

Regarding the independence of the recovery time from asynchronized periods, it is more obvious when we plot the required number of beating for the recovery from asynchronized periods to synchronized periods. As shown in Figure 7(b), all the length of asynchronized condition was within 30 beatings independent to cultivation time, whereas the length of synchronized condition varied from less than 10 beatings to more than 40 beatings.

**Figure 8:**
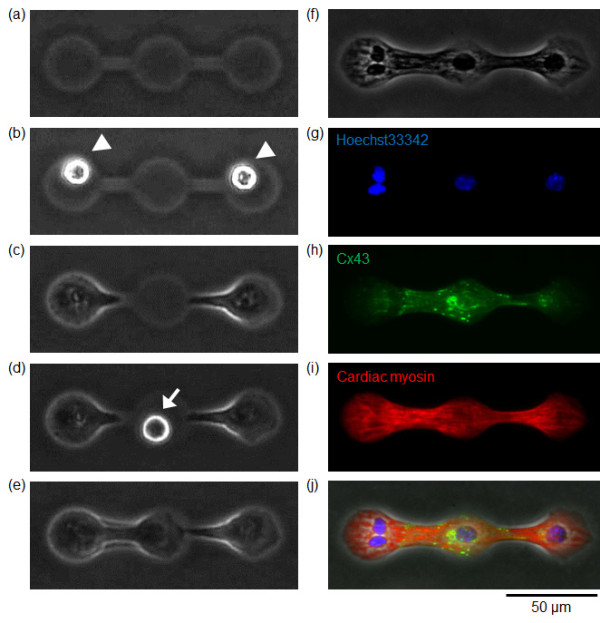
**Immunostaining of synchronized three cardiomyocyte network**. (a) - (e) Phase-contrast images of arrangement and cultivation process of three cardiomyocyte network. (a) Agarose microchamber. (b) Two cardiomyocytes set in both ends of the microchambers (white arrowheads). (c) Two cardiomyocytes having different beating rhythms was observed 1 day after cultivation started. (d) The third cardiomyocyte set in the center microchamber (white arrow). (e) After their physical contact, all three cardiomyocytes synchronized (12 h after recultivation started). (f) Phase-contrast image of three cardiomyocyte after fixation with 4% Formaldehyde solution. (g) - (i) Fluorescence images of (g) Nucleus (Hoechst33342; blue), (h) connexin-43 (green), (i) heavy chain cardiac myosin (red). (j) Phase-contrast image superimposed on the fluorescence images (g) - (i).

**Figure 9:**
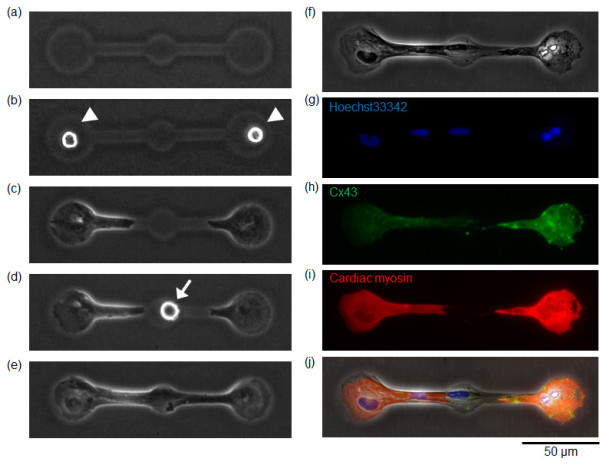
**Immunostaining of synchronized two cardiomyocytes connected by a fibroblast**. (a) - (e) Phase-contrast images of arrangement process of cardiomyocytes and a fibroblast. (a) Agarose microchamber. (b) Two cardiomyocytes set in both ends of the microchambers (white arrowheads). (c) Two cardiomyocytes with different beating rhythms were observed 3 days after cultivation started. (d) A fibroblast set at the center of the microchambers (white arrow). (e) Two cardiomyocytes were synchronized through the fibroblast (12 h after recultivation started). (f) Phase-contrast image of two cardiomyocytes connected by a fibroblast after fixation with 4% Formaldehyde solution. (g) - (i) Fluorescence images of (g) Nucleus (Hoechst33342; blue), (h) connexin-43 (green), (i) heavy chain cardiac myosin (red). (j) Phase-contrast image superimposed on the fluorescence images (g) - (i).

Regarding the electrical conductivity, Figures 8 and 9 shows the results of immunostainings of gap-junction proteins (connexin-43) to the three cardiomyocyte network (CM-CM-CM) and the two cardiomyocyte network connected by a fibroblast (CM-F-CM). As far as we can see in the Figures 8(h) and 9(h), at least connextin-43 was observed both in cardiomyocytes and fibroblasts in both of the networks. Summing up to the results of the direct measurement of electrical conductivity of CM-F-CM by patch-clamp measurement and immunostaining, electrical conductivity was maintained among CM-F-CM networks.

One possible reason for asynchronization occurrence might be the limited intercellular communication due to the lower electrical conductivity caused by the fibroblast-cardiomyocyte gap junctions [4,5], and another is the contribution of the mechanochemical coupling, i.e. mechanical stretching caused by the beating of neighboring cells triggers or enhances the calcium release in cardiomyocytes, induced synchronization tendency in cardiomyocyte network [23,24].

If the asynchronization is due to the lower electrical conductivity, the more fibroblasts are added between two cardiomyocytes, the less communication should be recorded between those cardiomyocytes. As shown in Table 1, the addition of fibroblast into the cardiomyocyte network decreased the propagation velocity, and is indicating the reduction of ability to respond to the cardiomyocytes. The addition of fibroblasts also lengthens the pathway between two cardiomyocytes, however, the propagation of signals did not changed caused by the length differences. That is, only the increase number of fibroblasts should influence the synchronization ability.

If, on the other hand, the synchronization is due to the mechanochemical coupling of neighboring cardiomyocytes, the contribution of physical contact and shorter distance of beating cardiomyocytes should be large to maintain the synchronization state in the cell network.

**Figure 10:**
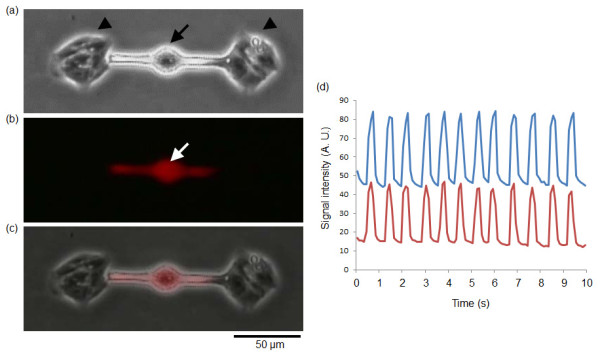
**Microinjection of fluorescence dye into a HeLa cell between synchronized two cardiomyocytes**. (a) Phase-contrast image of two cardiomyocytes (black arrowheads) beating synchronizaiton connected by a HeLa cell (black arrow). (b) Fluorescence image of Alexa Fluor 568 Hydrazide (white arrow) injected into the HeLa cell. (c) Phase-contrast image superimposed on the fluorescence image of (b). No dye transfer was found from the HeLa cell to the two cardiomyocytes. (d) Time course of synchronized beating rhythm of two cardiomyocytes connected by a HeLa cell. Bar, 50 μm.

To clarify and discuss more carefully about the contribution of above two factors for cardiomyocytes' synchronization and contribution of fibroblasts, we have then examined another three cell connection experiment. That is, two cardiomyocyte cells were connected by a HeLa cell. HeLa cells are one of the cell line and is not regarded as the connecting cells between cardiomyocytes. Figure 10(a) is the phase-contrast micrograph of the CM-HeLa-CM network. When we added the Alexa Fluor 568 Hydrazide into the HeLa cell and found no transportation of fluorescence dye to two cardiomyocytes (Figure 10(b)). And the two ends of cardiomyocytes continued synchronized as shown in Figure 10(d). The results indicate that the synchronization of two cardiomyocytes also could not only by the electrical conductivity like gap-junction connection nor cytoplasmic transportation.

For example, as described in our previous report [24], slight (sarcomere-level) displacement was enough to trigger synchronization as far as enough acceleration was generated. If it is correct, the existence of fibroblast might intercept acceleration conduction and reduced the stabilization of synchronization.

We have prepared the human induced pluripotent stem cell-derived cardiomyocytes and confirmed their fundamental ion-channel abilities [25], and we also have indicated the importance of control of community effect of cardiomyocytes, which can explain the difference of compound responses depending on cell network sizes [26]. Hence, considering the appropriate arrangement of fibroblasts among cardiomyocyte networks to represent re-modeling heart or aged heart, we might be able to improve on-chip *in vitro *cardiomyocyte network model for cardiotoxicity testing having more precise and sensitive human QT prolongation measurement.

## Conclusions

In this paper, as a part of our constructive/re-constructive approach to fabricate artificial higher complexity of cellular system, functional cell-network, we have examined the meaning and contribution of fibroblasts in the cardiomyocyte network using on-chip single-cell-based cultivation system. Our results summarized as (1) propagation velocity of electrophysiological signals between cardiomyocytes decreased depending on the increasing number of fibroblasts, not the lengths of fibroblasts; (2) fluctuation of interbeat intervals of synchronized two cardiomyocyte network connected by a fibroblast did not always decreased, and was different from homogeneous cardiomyocyte networks, and (3) the synchronized cardiomyocytes connected by fibroblasts loses their synchronized condition and recovered to synchronized condition, in which the length of asynchronized period was independent to their cultivation time whereas the length of synchronized period increased according to cultivation time. All above results indicated that the importance of the influence of the fibroblasts in a cardiomyocyte cluster from the viewpoint of synchronization, i.e., reduction of the ability of synchronization.

## Limitations of the study

The exact nature of the cell in the middle is not 100% clear but it is likely a fibroblast because this cell is fast cell division time, fast extension speed, and no staining of cardiomyocyte marker.

## Abbreviations

CCD: charge-coupled device; DMEM: Dulbecco's modified Eagle's medium; EDTA: ethylenediaminetetraacetic acid; PBS: Phosphate-buffered saline;VCR: video cassette recorder.

## Competing interests

The authors declare that they have no competing interests.

## Authors' contributions

TK and FN carried out whole experiments and participated in the design of the study and contributed to the drafting of the manuscript. KY conceived of the study, participated in its design and coordination and drafted the manuscript. All authors read and approved the final manuscript.
